# Pecan Shell-Derived Activated Carbon for High-Electrochemical Performance Supercapacitor Electrode

**DOI:** 10.3390/ma17133091

**Published:** 2024-06-24

**Authors:** Sarah J. Zou, Mumukshu D. Patel, Lee M. Smith, Eunho Cha, Sheldon Q. Shi, Wonbong Choi

**Affiliations:** 1 Department of Electrical Engineering, Stanford University, Stanford, CA 94305, USA; sjzou@stanford.edu; 2 Department of Materials Science and Engineering, University of North Texas, Denton, TX 76207, USA; mumukshupatel@my.unt.edu (M.D.P.); our_benefactor1016@hotmail.com (E.C.); 3 Department of Mechanical Engineering, University of North Texas, Denton, TX 76207, USA; leemiller.smith27@gmail.com

**Keywords:** activated carbon, pecan shell, supercapacitor, electrode

## Abstract

Carbon nanomaterials-based electric double-layer capacitors (EDLCs) are reliable and appealing energy-storage systems offering high power density and long cycling stability. However, these energy storage devices are plagued with critical shortcomings, such as low specific capacitance, inefficient physical/chemical activation process, and self-discharge of electrode materials, hindering their future application. In this work, we use a self-activation process, an environmentally benign and low-cost process, to produce high-performance activated carbon (AC). Novel activated carbon from pecan shells (PS) was successfully synthesized through a single-step self-activation process, which combines the carbonization and activation processes. The as-synthesized pecan shell-derived activated carbon (PSAC) provides a high-porosity, low-resistance, and ordered pore structure with a specific pore volume of 0.744 cm^3^/g and BET surface area of 1554 m^2^/g. The supercapacitors fabricated from PSAC demonstrate a specific capacitance of 269 F/g at 2 A/g, excellent cycling stability over 15,000 cycles, and energy and power density of 37.4 Wh/kg and of 2.1 kW/kg, respectively. It is believed that the high-efficiency PSAC synthesized from the novel self-activation method could provide a practical route to environmentally friendly and easily scalable supercapacitors.

## 1. Introduction

The increasing need for environmentally benign and sustainable energy solutions has spurred significant research into renewable energy storage systems [1]. Among the available energy-storage systems, supercapacitors, with their incredibly high power density, ultra-fast charging/discharging rate (<60 s), high cycle life (>100,000), and excellent stability compared to traditional capacitors and lithium-ion batteries, are an essential candidate to power future portable and flexible electronics, electric vehicles, and emergency door opening in airplanes [1,2,3]. To meet the demands of green energy capture and usage, supercapacitor performance needs to be improved [4]. Recent research has significantly improved the electrochemical performance of supercapacitors through the design and synthesis of novel carbon-based electrode materials [2,3]; however, these are often produced by non-renewable energy sources and involve environmentally hazardous processes. Therefore, further breakthroughs are needed to produce high-efficiency carbon materials by using renewable resources and an environmentally benign processes [5].

Over the past ten years, scientists have explored various carbon-based substances, including carbon nanotubes (CNTs), mesoporous carbon, graphene, and carbon nanofibers (CNFs), as potential materials for supercapacitors. This is attributed to their high specific surface area (SSA), electrical conductivity, and electrochemical stability. The existing commercial precursor for producing activated carbon originates from non-renewable energy sources. As an alternative, biomass-based ACs have been derived from nut shells including corn stalk core [6], coffee shell [7], hemp [8], human hair [9], and methylene blue [10]. Nutshells, which contains high amount of lignin and ordered cellular structure, have been widely utilized as a precursor for synthesizing large-scale activated carbon with a high product yield (>60%) [11,12]. Further, activated carbon from nutshells demonstrate excellent physical properties including high-surface area (>1000 m^3^/g) and an interconnected pore structure [6,7,8,9,10]. Similarly, pecan shells are an attractive candidate for biomass-activated carbon material because of its abundance—with 246 million pounds produced as agriculture waste per year in the United States. Since the amount of agricultural waste biomass being generated is predicted to increase with the continued growth of agricultural activities, it is important to generate useful products from these wastes. ACs from pecan shell have been investigated for use as a heavy metal ion water filter and carbon dioxide absorber [13,14,15]. However, the use of ACs from pecan shell as a supercapacitor electrode have not been reported. Further, few physical and chemical activation processes have been developed to synthesize activated carbon from nut shells; these methods involve toxic reagents such as KOH, ZnCl_2_, H_3_PO_4_, H_2_SO_4,_ and strong acids that are later released into the environment [15,16,17,18,19,20,21]. Recently, a self-activation process was developed as an alternative to these toxic physical and chemical processes for fabricating high performance activated carbon by our research group [22,23,24,25]. Self-activation utilizes the in situ gases generated during the heat treatment to activate the carbon in biomass [22]; since no additional activating agent was used, this self-activation process is environmentally benign and economical.

Here, we report an efficient synthesis and self-activation of the activated carbon (AC) from pecan shells for the application of supercapacitor electrode. The as-synthesized pecan shell activated carbon (PSAC) exhibited a high specific pore volume with narrow pore distribution, and high specific BET surface area. These appealing characteristics of the PSAC result in a high specific capacitance, high power density, and excellent cycling stability of the supercapacitor. The outstanding electrochemical performance of these PSACs electrodes could help meet the growing demand for sustainable and scalable fabrication of supercapacitors.

## 2. Experimental

### 2.1. Activation of Pecan Shells

Locally grown pecans were collected and shelled. The pecan shells were placed in an open ceramic crucible and introduced into a versatile box furnace (STY-1600C, Sentro Tech Corp., Strongsville, OH, USA). Subsequently, the furnace door was sealed, and a vacuum was applied until a pressure of −730 torr (96% vacuum) was reached. No inert gases were used in the activation during the self-activation process. Then, the furnace’s air outtake valve was sealed, preventing gas exchange with the external environment. The temperature was then increased with a maximum ramping rate of 10 °C/min to 1050 °C. At 1050 °C, the sample was left to dwell for either 5 h, 10 h, and 15 h. Hereafter, we refer to the samples with the heat treatment at set dwelling times as PSAC-5, PSAC-10, and PSAC-15, respectively. During this dwelling time the samples in the furnace underwent self-activation. After dwelling for the specified amount of time, the furnace was then cooled to room temperature at a rate of 10 °C/min, where the internal environment of the furnace was naturally cooled to ambient temperature. Then, they were removed from the furnace and small samples were taken for surface characterization. The rest were ground into a powder for further processing. 

### 2.2. Physical Characterization

We conducted nitrogen adsorption and desorption to measure the specific surface area, pore volume, and pore size distribution of the PSACs through the Brunauer–Emmett–Teller (BET) method (3-Flex 3500, Micromeritics Instrument Corp., Norcross, GA, USA) [24]. The PSAC sample was placed into a test tube and degassed at 350 °C in a degasser for one day (VacPrep 061, Micromeritics Instrument Corp.). Nitrogen adsorption and desorption experiments were conducted at various relative pressures over the course of one day to measure the surface area of the PSAC. The assessment of mesopores-macropore surface area and pore volume was performed using the Barrett–Joyner–Halenda (BJH) method (3-Flex 3500, Micromeritics Instrument Corp.). Additionally, micropore surface area was assessed by the Horvath–Kawazoe (HK) method, and micropore pore volume was calculated by t-plot. Raman spectroscopy (Nicolet Almega XR Dispersive Raman spectrometer, Thermo Scientific, Waltham, MA, USA)) was used to characterize the carbon peak on the compressed powder PSAC samples at a range of 1000–3000 cm^−1^. Morphologies of the as-fabricated PSAC were measured and assessed by using scanning electron microscope (FEI Nova-NanoSEM 230, Field Electron and Ion Company, Hillsboro, OR, USA). Microstructure investigation was performed through transmission electron microscopy (Philips EM420 TEM, Philips, Amsterdam, The Netherlands).

### 2.3. Electrochemical Characterization

Utilizing N-Methyl-2-pyrrolidone (NMP) as the solvent, we combined activated carbon powder samples with polyvinylidene fluoride (PVDF) and carbon black at a weight ratio of 80:10:10 (AC: PVDF: carbon black). The mixtures were stirred with a magnetic stirrer to achieve a homogeneous slurry, and the resulting activated carbon slurry was applied onto a nickel foam current collector. The electrodes were assembled into a symmetric coin cell with a polypropylene surfactant-coated separator (Celgard 3501) and 6 M KOH as electrolyte. Electrochemical assessments were carried out using a potentiostat, specifically the Gamry Reference 3000. These assessments included cyclic voltammetry (CV) and galvanostatic charge–discharge (GCD) to calculate specific capacitance and capacity. Additionally, electrochemical impedance spectroscopy (EIS) was conducted to evaluate impedance characteristics. The specific capacitance obtained from cyclic voltammetry was computed using the following Equation (1) [26]:

(1) $$C_{S}=\frac{4\int I\;dv}{um\Delta V}$$

where *u* is the scan rate (mV/s), *m* is the total mass of the two electrodes (g), and ΔV denotes voltage window (V). 

We can also calculate specific capacitance from galvanostatic charge–discharge graphs at specific discharge current *I*, discharge time Δ*t* [2]:

(2) $$C_{S}=\frac{4I\Delta t}{m\Delta V}$$

From specific capacitance, energy (Wh/kg) and power densities (W/kg) were calculated using the following Equations (3) and (4) given a voltage window *V*, and discharge time *t*:

(3) $$E=\frac{1}{2}C_{s}V^{2}$$

(4) $$P=\frac{E}{t}$$

## 3. Results and Discussion

### 3.1. Physical Characteristics

**Figure 1:**
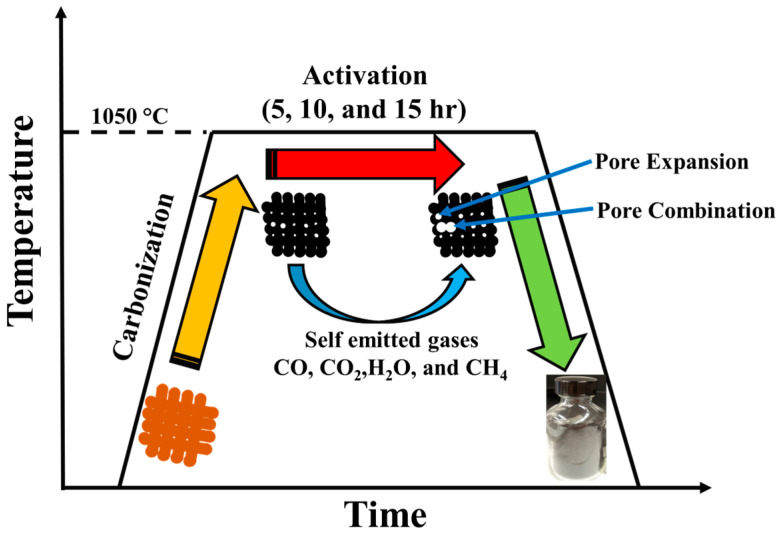
Schematic and the corresponding temperature vs. time graph outlining the self-activation process of the pecan shell. The middle segment represents dwelling temperature and time.

**Figure 2:**
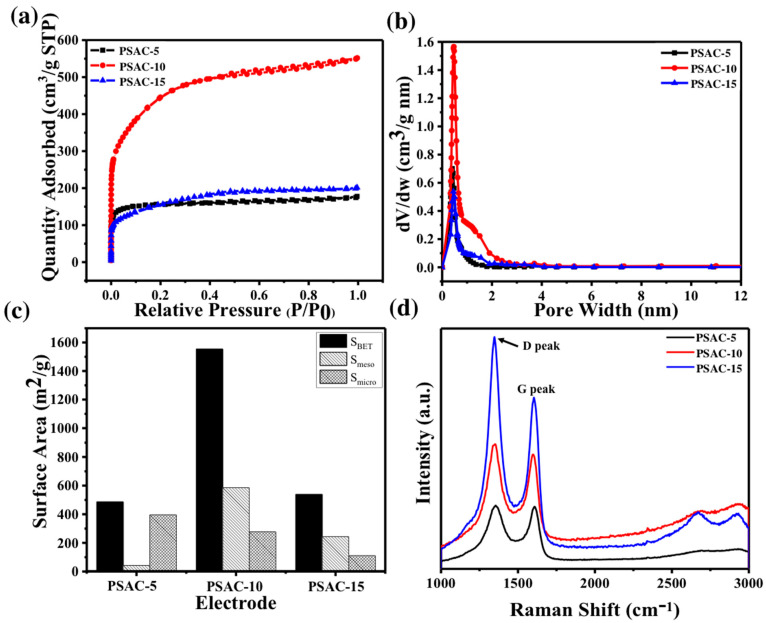
(**a**) Nitrogen adsorption–desorption for PSAC-5, 10, and 15; (**b**) pore size distribution for PSAC-5, PSAC-10, PSAC-15; (**c**) surface area comparisons between PSAC-5, PSAC-10, and PSAC-15; (**d**) Raman spectra of PSAC-5, PSAC-10, PSAC-15.

The details of the carbonization and activation process for the pecan shell are demonstrated in Figure 1. The emitted gas from carbonization of the pecan shell at a temperature of ~1050 °C may serve as activating agents. These gaseous agents are identified as self-emitted gases for the activation of the carbonization process. The nitrogen (N_2_) adsorption–desorption isotherms at 77 K of the PSAC of 5, 10, and 15 h of activation time are shown in (Figure 2a). The isotherm curves (Figure 2a) exhibited concave isotherm type I (according to IUPAC), indicating that the PSAC are microporous solids [27]. As P approached P_0_, the specific surface area of the PSAC increased minimally. The amount of gas adsorbed by PSAC-15 and PSAC-5 is very low, indicating low specific surface area. The BET specific surface areas of PSAC-5, PSAC-10, and PSAC-15 measured were 486.4 m^2^/g, 1553.6 m^2^/g, and 538.7 m^2^/g, respectively (Figure 2c). When activation time was increased from 5 to 10 h, the specific surface area tripled, which is attributed to pore expansion. The resulting decrease in surface area when the activation time changes from 10 to 15 h can be ascribed to the overexpansion of pores leading to breaking of cell walls, reducing pore volume and surface area [22]. The measurement of pore size distribution and specific pore volume (SPV) is calculated by density functional theory (DFT) model as illustrated by Figure 2b. Despite the different activation times, the pore size distribution for all three samples were similar in their high concentration of micropores (width < 2 nm) as seen in Table 1. The PSAC-10 demonstrated the widest pore size distribution as compared to others (PSAC-5 and PSAC-15). Raman spectra (Figure 2d) of the PSACs showed prominent D (1335 cm^−1^) and G (1590 cm^−1^) bands. The D band is a result of the disordered or defected carbon structure in the activated pecan shell carbon, while G band represents graphite-like structure.

The I_D_/I_G_ for PSAC-5, PSAC-10, and PSAC-15 are 1.04, 1.181, and 1.471, respectively. The high degree of disorderness of PSAC-15 can be attributed to its long activation time and is further supported by the emergence of 2D peak (~2600 cm^−1^), representing the degree of crystallinity and graphene structure [28,29].

**Figure 3:**
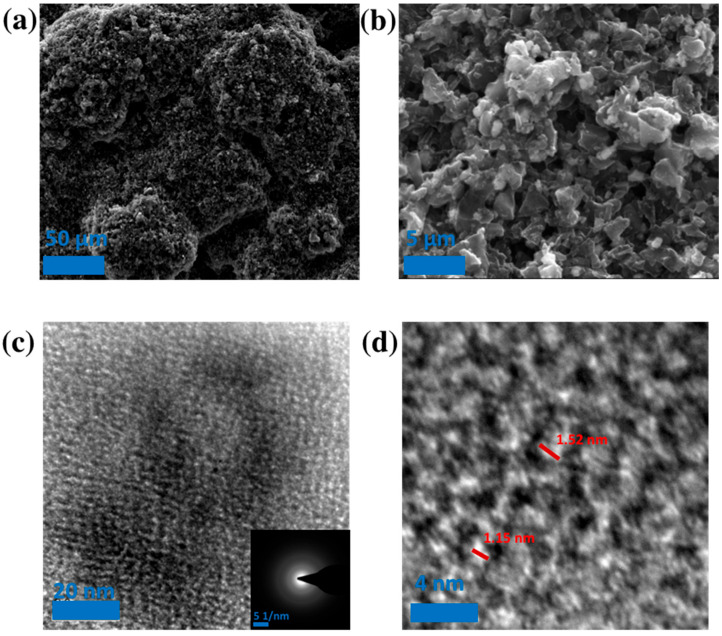
SEM and TEM Images of PSAC-10: (**a**,**b**) SEM image of PSAC-10 interconnected pore structure; (**c**) 100 nm width TEM displays amorphous structure that is confirmed with inset; (**d**) 10 nm width TEM showing micropore structure.

The SEM images of the PSAC-10 are presented in Figure 3a,b for investigating the morphology and pore structure of the ACs. A TEM image of the PSAC-10 is shown in Figure 3c,d, where the disordered amorphous carbon structure of PSACs is clearly seen. This result was further confirmed by the diffraction patterns in the inset of Figure 3c and is consistent with the Raman analysis [30]. A uniform distribution of micropores with a pore size of < 2 nm for the PSAC is shown in Figure 3d, which support the pore size distribution result as determined by BET method (Figure 2b).

### 3.2. Electrochemical Performance

**Figure 4:**
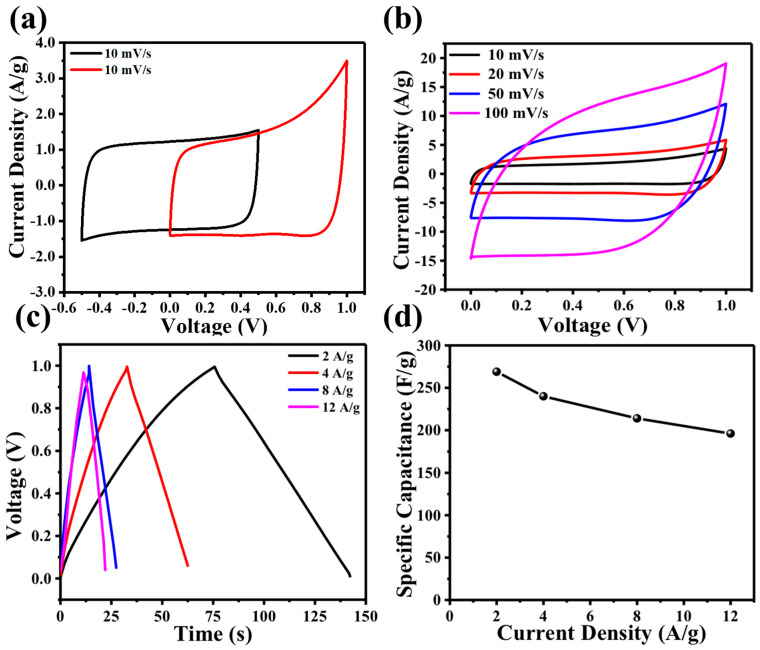
(**a**) Cyclic voltammetry of PSAC-10 across voltage windows of −0.5–0.5 and 0–1.0 V; (**b**) cyclic voltammetry of PSAC-10 supercapacitor in 6 M KOH with voltage window of 0–1.0 V and scan rates of 10 mV/s, 20 mV/s, 50 mV/s, and 100 mV/s; (**c**) galvanostatic charge–discharge profile of PS-10 supercapacitor with current density of 2 A/g, 4 A/g, 8 A/g, and 12 A/g in a voltage range of 0–1.0 V; (**d**) specific capacitance plotted against current density calculated from GCD profiles.

Thorough analysis of the electrochemical characteristics of PSAC involved the application of cyclic voltammetry (CV), galvanostatic charge–discharge (GCD), and electrochemical impedance spectroscopy (EIS). These assessments were carried out within a two-electrode coin cell configuration, employing a 6 M KOH electrolyte solution. Only the electrochemical performance of PSAC-10 was evaluated as it exhibited superior physical properties compared to PSAC-5 and PSAC-15. Figure 4a shows cyclic voltammetry at 10 mV/s scan rate in different voltage windows. Figure 4b illustrates the cyclic voltammetry (CV) curves of PSAC-10, showcasing scan rates ranging from 10 to 100 mV/s. The well-defined rectangular characteristics with no redox peaks show the EDLC supercapacitor behavior of PSAC-10. At a high scan rate of 100 mV/s, the electrode material exhibited a slight departure from the ideal rectangular shape with no faradic reaction peaks which indicates the electrode’s ability to facilitate rapid ion transfer and diffusion [31]. The excellent reversibility of the electrode is evident in the diagonal symmetry observed in the rectangular cyclic voltammetry (CV) curve [32,33]. The satisfactory supercapacitor performance of PSAC-10 can be attributed to the balanced ratio of micropores and optimally sized mesopores (2–5 nm) [34].

Galvanostatic charge–discharge profiles (Figure 4c) show typical symmetric linear triangular curves with minimal IR drops at all current densities of 2, 4, 8, and 12 A/g. The minimal IR drop for all current densities reveals PSAC-based electrodes to have low internal resistance. The calculated specific capacitances (Figure 4d) from the discharge curves are 269, 240, 214, 196 F/g at current densities 2, 4, 8, 12 A/g, respectively. The specific capacitance of PSAC-10 was only reduced by 27% when current density is increased from 2 A/g to 12 A/g.

**Figure 5:**
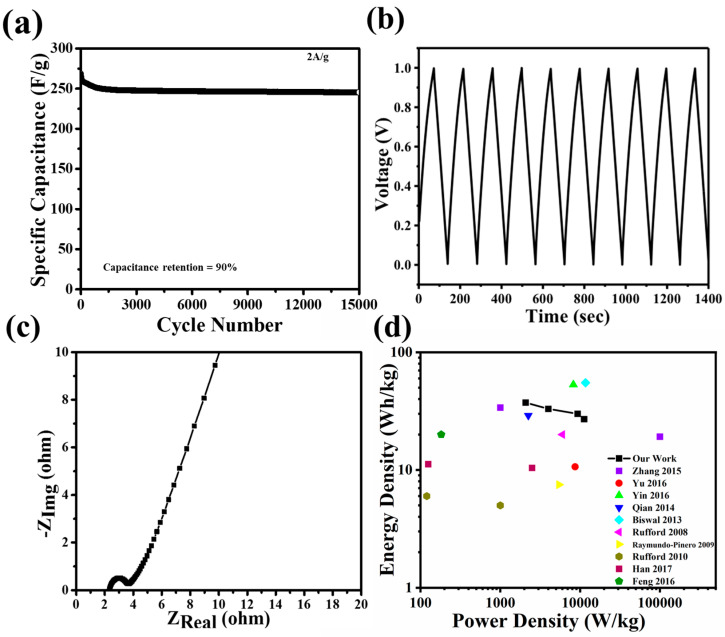
(**a**) Specific capacitance of PSAC-10 supercapacitor over 15,000 charge–discharge cycles at a current density of 2 A/g; (**b**) galvanostatic charge–discharge profile of PSAC-10 supercapacitor with current density of 2 A/g; (**c**) electrochemical impedance spectroscopy of Nyquist plot of PSAC-10 supercapacitor with equivalent circuit as inset; (**d**) Ragone plot of energy and power density values of PSAC-10 in compared with other reported works at 1 V working voltage [9,27,39,40,41,42,43,44,45,46].

Figure 5a,b show the long-term cyclability of the PSAC-10 for 15,000 cycles at a current density of 2 A/g demonstrating excellent capacity retention of 90% with an average coulombic efficiency of 97%. The high performance of PSAC-10 can be attributed to its high surface area of 1554 m^2^/g and pore volume of 0.74360 cm^3^/g (0.44459 cm^3^/g for micropore and 0.28698 cm^3^/g mesopores). Consequently, the high percentage of micropore and mesopores makes the material optimal for the efficient adsorption of electrolytic ions i.e., K^+^ and OH^−^ ions [35,36]. It is to be noted that K^+^ has a bare ionic size of 1.33 Å (0.133 nm) and a hydrated ion size of 3.31 Å (0.331 nm) while OH^−^ has a bare ionic size of 1.76 Å (0.176 nm) and a hydrated ion size of 3.00 Å (0.300 nm) [35].

Electrochemical impedance spectroscopy was conducted to measure the internal and overall cell resistance (Figure 5c). The equivalent circuit represents the electrochemical cell (Figure 5c inset). The intercept of x-axis in Nyquist plot represents ohmic resistance (R_s_) in the high-frequency range, which arises from electrolyte, current collector, and activated carbon film [37]. The ohmic resistance of ~2 Ω suggests excellent electrical conductivity and good quality of as-fabricated activated carbons. The semi-circle appearing in medium frequency range exemplify R_c_C_c_ unit attributing to charge transfer resistance (~2 Ω) at the electrode/electrolyte resistance [38]. The straight line in low frequency range is ascribed to capacitance (C).

In comparison with previous studies [9,27,39,40,41,42,43,44,45,46] on biomass-derived activated carbons for supercapacitors, PSAC-10 demonstrated consistently higher energy density and power density calculated at a range of current densities such as 2, 4, 8, and 12 A/g, as shown in Figure 5d. This is due to the unique self-activation process and pecan shell precursor structure. Notably PSAC-10 achieved an energy density of 36 Wh/kg and power density of 2078 W/kg while also maintaining almost all initial capacitance through 15,000 cycles.

## 4. Conclusions

The as-fabricated pecan shell-derived activated carbon (PSAC) was synthesized by an environmentally benign self-activation process, and the electrochemical performance of PSAC as supercapacitor electrodes was evaluated. The PSAC, fine-tuned under varied activation times of 5, 10, and 15 h, demonstrated outstanding physical characteristics, boasting a high specific surface area (SSA) of 1554 m^2^/g and a specific pore volume (SPV) of 0.74360 cm^3^/g. After 10 h of activation, the PSAC-10 exhibits an optimal combination of micro and meso pores, enabling efficient adsorption by offering electroactive sites. This leads to a specific capacitance of 269 F/g at a current density of 2 A/g, accompanied by an impressive cycling stability of around 93% after 15,000 cycles. Furthermore, PSAC-10 maintained 73% of its capacitance at current density 2 A/g when current density was increased to 12 A/g. This straightforward method for creating activated carbon from pecan shells via a novel self-activation method with strong electrochemical capabilities could provide a practical route to environmentally friendly and easily scalable supercapacitor production.

## Figures and Tables

**Table 1:** Table of BET specific surface area (SSA), pore volume, and pore size distribution of the PSACs.

Sample	BET SSA	SPV (cm^3^/g)			
	(m^2^g^−1^)	Micropore	Mesopores	Macropore	Total
PSAC-5	486.1	0.20433 (86.8%)	0.02378 (10.1%)	0.00739 (3.14%)	0.23550
PSAC-10	1553.6	0.44459 (59.8%)	0.28698 (38.6%)	0.01203 (1.62%)	0.74360
PSAC-15	540.8	0.19096 (60.7%)	0.11693 (37.2%)	0.00681 (2.2%)	0.31470

## Data Availability

Data are contained within the article.
